# Supplementary material for: Usability and psychometric properties of a battery of tools to assess intelligence, executive functioning, and sustained attention in Tanzanian children
Source: PLoS One. 2024 Dec 30;19(12):e0315058. doi: 10.1371/journal.pone.0315058 (PMC11684700; doi:10.1371/journal.pone.0315058)

**S1 APPENDIX**

**Usability and psychometric properties of a battery of tools to assess intelligence, executive functioning, and sustained attention in Tanzanian children**

Georg Loss^1,2*^, Hannah Cummins^3^, Nicolaus Gutapaka^4^, Jane Nyandele^4^, Sylvia Jebiwott ^3^, Deborah Sumari^4^, Thabit Athuman ^4^, Omary Juma^4^, Susanne P. Martin-Herz^5^, Ally Olotu^4^, Michelle S. Hsiang^3,5,6^, Günther Fink^1,2^

Author affiliations:

1 Swiss Tropical and Public Health Institute, Switzerland

2 University of Basel, Switzerland

3 Malaria Elimination Initiative, Institute of Global Health Sciences, UCSF, U.S.A.

4 Ifakara Health Institute, Biomedical Research and Clinical Trials Department, Tanzania

5 Department of Pediatrics, UCSF, U.S.A.

6 Department of Epidemiology and Biostatistics, UCSF, U.S.A.

* Corresponding author

E-mail: georg.loss@swisstph.ch

**Table s1: Household listing and random selection by age year group**

**Table s2: List of household assets**

**Figure s1: Study sample flow chart**

**
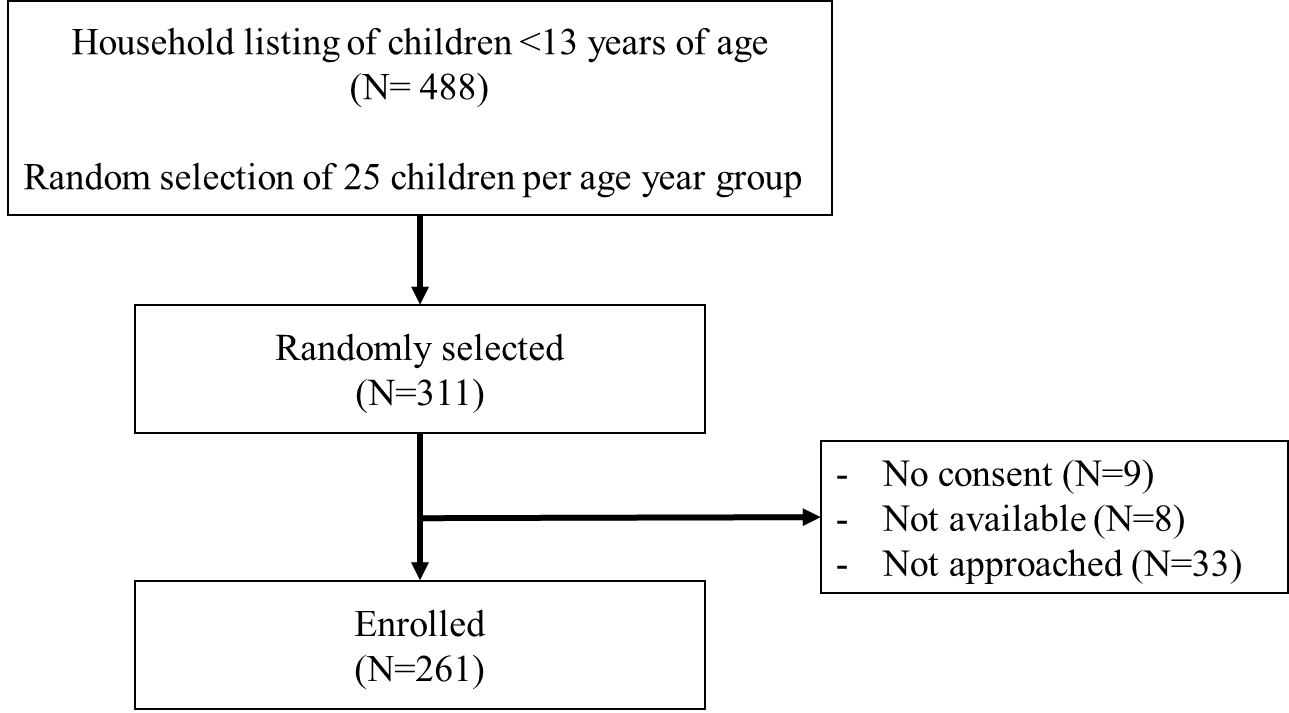
**

No difference in ages between groups i) enrolled, ii) no consent, iii) not available, iv) not approached were found using ANOVA.

Due to reduced internet connectivity in the data collection areas and resulting delays in database updates, one additional participant was enrolled and included in the study, bringing the total to N=261 instead of the planned N=260.

**Figure s2: Study sample height-for-age z-score distribution (and standard normal distribution in orange)**


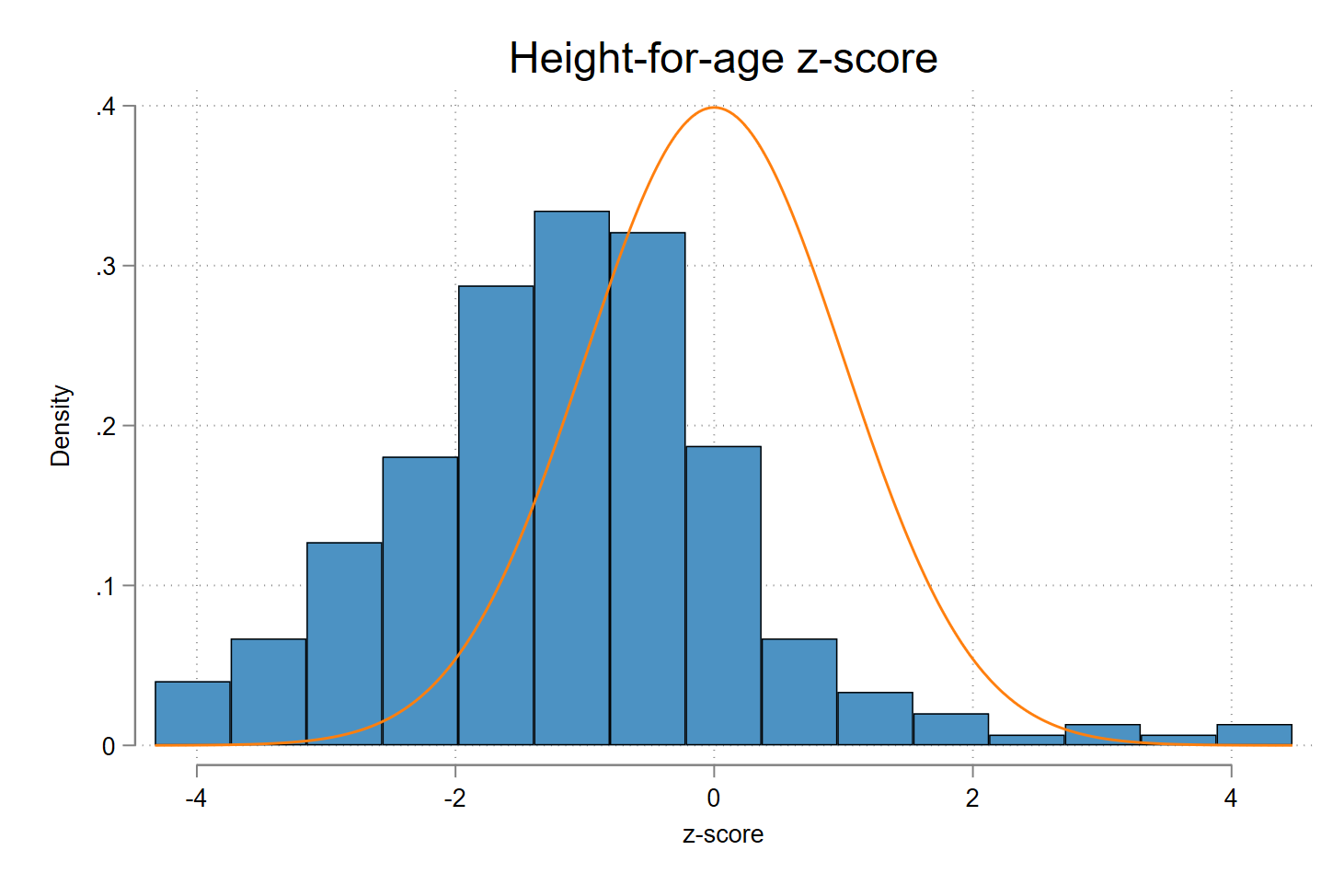


**Figure s3: Height-for-age z-score by age year group**


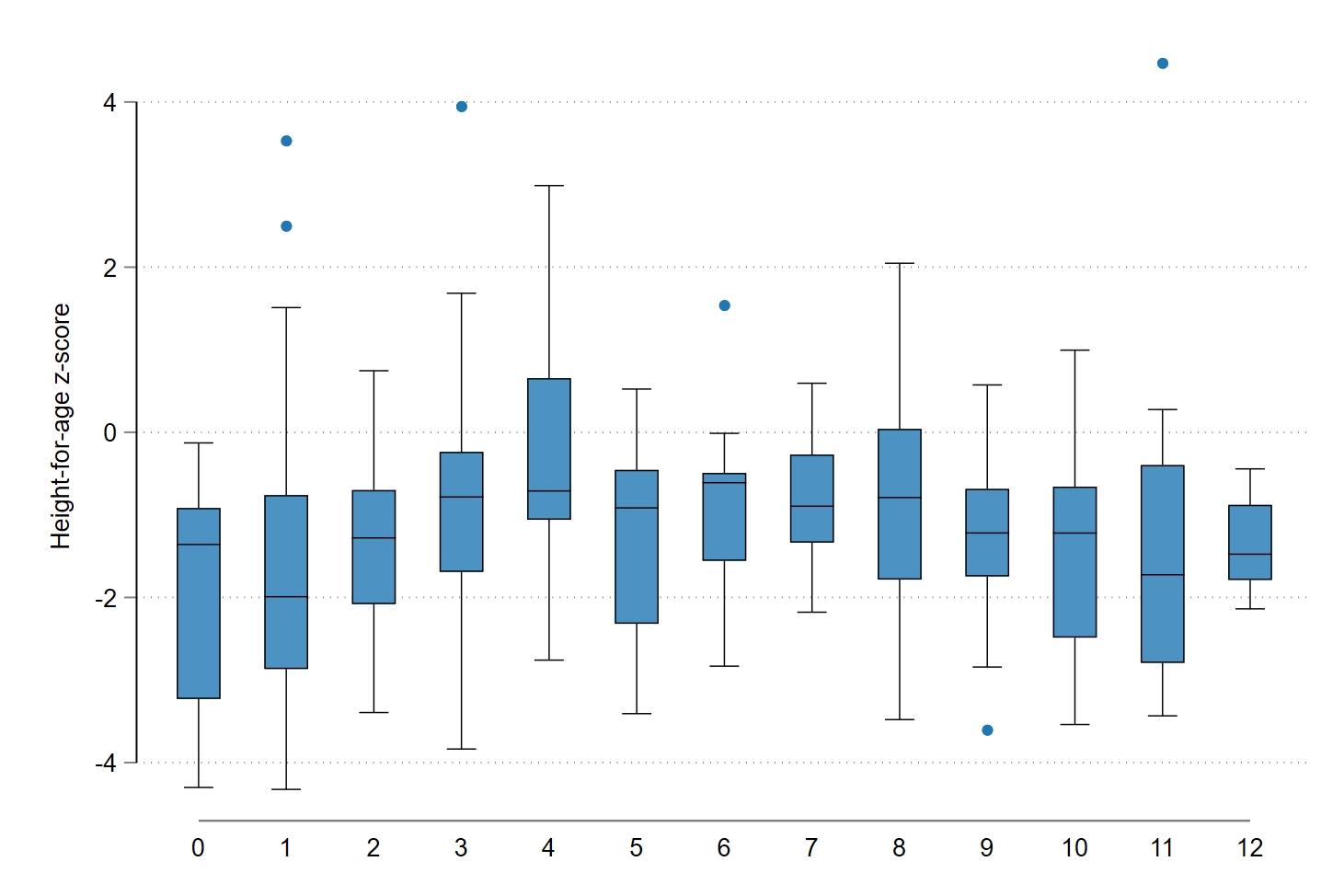

Supplement: S1 Appendix — (DOCX) [file pone.0315058.s002.docx]
