# Supplementary material for: Usability and psychometric properties of a battery of tools to assess intelligence, executive functioning, and sustained attention in Tanzanian children
Source: PLoS One. 2024 Dec 30;19(12):e0315058. doi: 10.1371/journal.pone.0315058 (PMC11684700; doi:10.1371/journal.pone.0315058)
Supplement: Attachment — Submitted filename: response to reviewers.docx [file pone.0315058.s004.docx]

Randall Waechter

Academic Editor

PLOS ONE

**REBUTTAL LETTER,** PONE-D-24-26872

**CONCERNING**: PONE-D-24-26872, Usability and psychometric properties of a battery of tools to assess intelligence, executive functioning, and sustained attention in Tanzanian children

Dear Randall Waechter,

Thank you for the kind comments. We have done our best to address all comments and concerns raised by the academic editor and reviewers.

We submitted a change-tracked copy of our manuscript document highlighting any changes made to the original submission, as well as an unmarked version of our revised paper without tracked changes.

Please find our point by point response in the rebuttal letter below, editors’/reviewers’ comments in italic and our corresponding response in bold face.

**RESPONSE TO REVIEWERS:** PONE-D-24-26872

**JOURNAL REQUIREMENTS:**

*1. Please ensure that your manuscript meets PLOS ONE's style requirements, including those for file naming. The PLOS ONE style templates can be found at*

*https://journals.plos.org/plosone/s/file?id=wjVg/PLOSOne_formatting_sample_main_body.pdf and*

*https://journals.plos.org/plosone/s/file?id=ba62/PLOSOne_formatting_sample_title_authors_affiliations.pdf*

**AUTHOR RESPONSE: We update the formatting to meet PLOS ONE's style requirements following the listed style templates. Changes are indicated in the change-tracked main manuscript document. Specifically, we changed the appearance of headers, and figure captions, citations and file naming. We used PACE to check figures originally submitted. We originally submitted Figure 3 as .pdf file, and now submitted it as .tif file (named “fig_3.tif”). We adjusted dimensions for Figure 1 and 2 .eps figure files using PACE.**

*2. Please include a complete copy of PLOS’ questionnaire on inclusivity in global research in your revised manuscript. Our policy for research in this area aims to improve transparency in the reporting of research performed outside of researchers’ own country or community. The policy applies to researchers who have travelled to a different country to conduct research, research with Indigenous populations or their lands, and research on cultural artefacts. The questionnaire can also be requested at the journal’s discretion for any other submissions, even if these conditions are not met. Please find more information on the policy and a link to download a blank copy of the questionnaire here: https://journals.plos.org/plosone/s/best-practices-in-research-reporting. Please upload a completed version of your questionnaire as Supporting Information when you resubmit your manuscript.*

**AUTHOR RESPONSE: We completed a copy of PLOS’ questionnaire on inclusivity in global research. We submit it as supporting information file with the revised manuscript. Following the inclusivity questionnaire guidance, we added information on local approvals there and in the main manuscript’s ethics section, i.e. we added this statement in line 283 of the change-tracked main manuscript text: “*The study was approved by the President's Office, Regional Administration and Local Government Tanzania (PO-RALG). Additional information regarding the ethical, cultural, and scientific considerations specific to inclusivity in global research is included in the Supporting Information (S2 Inclusivity in global research checklist).*”**

*3. Thank you for stating the following financial disclosure: This work was supported by NIH/NIAID grant # 1U01AI155315-01A1.*

*Please state what role the funders took in the study. If the funders had no role, please state: ""The funders had no role in study design, data collection and analysis, decision to publish, or preparation of the manuscript.""*

*If this statement is not correct you must amend it as needed.*

*Please include this amended Role of Funder statement in your cover letter; we will change the online submission form on your behalf.*

**AUTHOR RESPONSE: Thank you for pointing this out. We kindly request to amend our funding statement as follows (also stated in the revision cover letter): "*This work was supported by NIH/NIAID grant # 1U01AI155315-01A1. The funders had no role in study design, data collection and analysis, decision to publish, or preparation of the manuscript*".**

*4. We note that you have included the phrase “data not shown” in your manuscript. Unfortunately, this does not meet our data sharing requirements. PLOS does not permit references to inaccessible data. We require that authors provide all relevant data within the paper, Supporting Information files, or in an acceptable, public repository. Please add a citation to support this phrase or upload the data that corresponds with these findings to a stable repository (such as Figshare or Dryad) and provide and URLs, DOIs, or accession numbers that may be used to access these data. Or, if the data are not a core part of the research being presented in your study, we ask that you remove the phrase that refers to these data.*

**AUTHOR RESPONSE: Thank you very much for pointing out this inconsistency.**

**The paragraph containing the “data now shown” expression, was referring to results detailed in Table 3, except this additional linear regression result in the original submission “*IDELA scores were however related to children being in kindergarten or pre-school vs. not (linear age adjusted increase in IDELA score standard deviations: beta, 95%CI, P-value: 0.64, 0.21-1.06, 0.004; data not shown)*.”**

**In this original submission statement, we inappropriately intended to inform the reader that this results is not detailed in Table 3 using “data not shown”. Thus, the term “data not shown” is in fact not accurate, as we show the linear regression coefficient here as a stand-alone result, and “data not shown in Table 3” would be more accurate.**

**To simplify, we now removed the “data not shown” (line 357 in the change-tracked manuscript) and added a reference to Table 3 in the following sentence (line 358) to this is a finding not detailed in Table 3.**

*5. Please include captions for your Supporting Information files at the end of your manuscript, and update any in-text citations to match accordingly. Please see our Supporting Information guidelines for more information:* [*http://journals.plos.org/plosone/s/supporting-information*](http://journals.plos.org/plosone/s/supporting-information)*.*

**AUTHOR RESPONSE: We have corrected the supplemental information in-text-citations to match the journal style. We have renamed and re-submitted the originally submitted supporting information document as “S1 APPENDIX”. We also added the “inclusivity in global research checklist” as supporting information file “S2” and added this statement in line 283 of the main manuscript text: “Additional information regarding the ethical, cultural, and scientific considerations specific to inclusivity in global research is included in the Supporting Information (S2 Inclusivity in global research checklist).” We further listed the Supporting Information documents at the end of the main body manuscript.**

*6. Please review your reference list to ensure that it is complete and correct. If you have cited papers that have been retracted, please include the rationale for doing so in the manuscript text, or remove these references and replace them with relevant current references. Any changes to the reference list should be mentioned in the rebuttal letter that accompanies your revised manuscript. If you need to cite a retracted article, indicate the article’s retracted status in the References list and also include a citation and full reference for the retraction notice.*

**AUTHOR RESPONSE: Thank you for your feedback. We have reviewed our reference list and found none of the cited papers have been retracted. No changes were made to the reference list as a result. Please let us know if you had concerns about any of the listed references and we will address this accordingly, and remove any retracted articles.**

*Additional Editor Comments:*

*Apologies for the long delay in responding to this submission. Finding reviewers for this manuscript was a significant challenge. I am pleased to report that both reviewers have recommended publication of the manuscript in PLOS ONE, pending a response to one question from one of the reviewers. For this reason, I have recommended Minor Revision. It should not take long to respond to the reviewer's query. This is an important area of work that the International Neuropsychological Society has recently recognized. The INS has called for more effort to both adapt measures for use in low-middle-income country settings and to develop culturally-appropriate measures from within LMICs. The manuscript will make an important contribution to this discussion.*

**AUTHOR RESPONSE: Thank you very much for your thoughtful review and recommendation regarding our manuscript submission to PLOS ONE. We appreciate your efforts in finding suitable reviewers and are grateful for their positive feedback.**

**We are pleased to hear that both reviewers have recommended our work for publication, pending our response to the remaining query. We are delighted that our manuscript may soon contribute to this important field highlighted by the INS.**

**REVIEWERS' COMMENTS TO THE AUTHOR:** PONE-D-24-26872

***REVIEWER #1:***

*I thought this was a well-designed feasibility study that was relevant to the larger goal of the researchers.*

*I did appreciate that the authors undertook this study to determine the appropriateness of non-local neurocognitive measures for the CHILD study and I think their foresight on the matter should be commended.*

**AUTHOR RESPONSE: Thank you very much, we appreciate the reviewer’s time and critical appraisal.**

*One question: What were some of the "similarities" you considered relevant between Yombo and Fukayosi/Kiwanga?*

**AUTHOR RESPONSE: Thank you for highlighting this. We added shared characteristics of these wards in this updated text passage (changes underlined) at the beginning of the methods section, line 81-86 of the change-tracked main manuscript:**

**“*This is a cross-sectional validation study conducted in Yombo, Tanzania in 2023. We selected the Yombo ward for its proximity and similarities to the CHILD study area, which is conducted in the adjacent Fukayosi and Kiwangwa wards of the same low malaria transmission region. Yombo shares several relevant characteristics with Fukayosi and Kiwangwa, including demographic makeup, rural settings, socio-economic conditions, and malaria transmission rates. Yombo is rural, consisting of 31 Hamlets with an estimated total population of 11,102 based on the most recent 2022 census*.”**

***REVIEWER #2:***

*The manuscript appears to be technically sound. The five selected instruments (along with pre-testing to assess their applicability and adaptations to fit the study’s context), appear suitable for assessing constructs of general intelligence, executive functioning, and sustained attention in Tanzanian children.*

**AUTHOR RESPONSE: Thank you very much, we appreciate the reviewer’s time and critical appraisal.**
